# Editorial: Best practices in clinical research conduct in veterinary medicine

**DOI:** 10.3389/fvets.2024.1533052

**Published:** 2024-12-10

**Authors:** Tracy L. Webb, Sarah A. Moore, Benjamin M. Brainard

**Affiliations:** ^1^Department of Clinical Sciences, Colorado State University, Fort Collins, CO, United States; ^2^BluePearl Science, Tampa, FL, United States; ^3^Department of Small Animal Medicine & Surgery, University of Georgia, Athens, GA, United States

**Keywords:** veterinary, clinical research, consensus statement, biobanking, recruitment, informed consent, naturally occurring disease, translational research

The importance of clinical research in improving health outcomes cannot be overstated. However, research design and reports must be of high quality to optimize potential benefits. Veterinary clinical researchers have a moral obligation to perform quality, impactful studies, and targeted research to define best practices in clinical research will promote continued improvement in this space. Such investigations highlight the importance of topics including enrolling a diverse patient population (1, 2), obtaining informed consent (3, 4), adverse event criteria (5), and reporting practices (6, 7) leading to the generation of more clinically and translationally relevant results.

Interest in and organization around the conduct of veterinary clinical research is increasing (8) and has the potential to play a significant role in improving patient outcomes. The publication of high-quality veterinary research facilitates the use of data in translational contexts to address unmet medical needs of animals and humans. The importance of model selection and benefits of studying naturally occurring disease in target populations (as opposed to studying induced disease under artificial circumstances) is increasingly recognized (9–14). However, less information is currently available about best practices in veterinary clinical research conduct than what is available in human medicine. Although there is overlap in many areas of clinical research between human and veterinary medicine, there are also significant differences such as the legal status of the patient, regulatory requirements (15), and the role of humane euthanasia and financial decisions on study outcomes.

This Research Topic was initiated to encourage and support researchers performing needed investigation into the conduct of veterinary clinical research. There are many emerging impactful topics and knowledge gaps in this area such as research regulation and reporting practices, incorporation of adaptive design and artificial intelligence (16), support of multi-site and collaborative projects, equity and diversity in clinical trials, methods of assessing research impact, and sustainable research practices. Included articles investigate the impact of consensus statements, opportunities to improve veterinary biobanking, best practices in study recruitment, and new regulatory guidelines on informed client consent.

Consensus statements play a key role in medical practice. They represent the combined recommendations of experts in the field, usually when there is a lack of comprehensive evidence available on a topic, and serve to translate available research findings into clinical practice recommendations. Although consensus statements have limitations and biases that should be considered, they can significantly influence patient treatment, health policy, and societal behavior (17). The impact of consensus statements on veterinary prescribing habits in clinical practice has not been fully evaluated, and Sainz et al. investigated the prevalence and appropriateness of omeprazole prescriptions in dogs at a veterinary teaching hospital before and after the publication of the 2018 American College of Veterinary Internal Medicine consensus statement on the rational administration of gastrointestinal protectants (18). The retrospective study compared the prescribing habits for the proton pump inhibitor omeprazole in dogs at an academic veterinary teaching hospital in the 12 months before and after the publication of the consensus statement. Although a small study, the results support the impact of the consensus statement on prescribing patterns for omeprazole in an academic veterinary hospital. Further investigation is needed to evaluate the impact of consensus statements in other practice environments and into other mechanisms to support appropriate medication prescription practices.

Sample biobanking facilitates impactful research through collection, cataloging, and storage of control and diseased biological samples allowing timely sample analysis. However, biobanking has been associated with controversy (19), and lack of best practices led to a New York Times best-selling book and movie about the generation of HeLa cells—used widely in research—without patient consent (20). Veterinary biobanks support both human and veterinary clinical research, storing biological samples from animals with naturally occurring disease for future use and distribution to academic researchers and industry. However, informed consent provided by owners for pets contributing to biobanks can be complicated by limited understanding of goals, purpose, and logistics of biobanking. McEnhill et al. performed a survey-based study to investigate pet owner perspectives, motivators, and concerns about veterinary biobanking with the goal of identifying opportunities to improve education, awareness of veterinary biobanking initiatives, and consent processes. Study results suggest veterinary biobanking initiatives are well received by owners, most of whom are willing to allow their pets to participate, and respondent concerns represent opportunities for veterinary biobanks to improve messaging and dissemination of results from work they support.

A successful clinical trial requires participants, but many factors can impede effective study recruitment. Quigley et al. provide guidelines on best practices for recruitment of patients to veterinary clinical trials. Strategies for recruitment of high-quality subjects for clinical research should utilize a holistic view of recruitment encompassing study design and logistics, representative participation, incentives, personnel resources, advertising, and participant retention. Although human clinical trial resources can provide guidance, effort also needs to be put into evaluating current practices and opportunities for process improvement that are specific to veterinary clinical trials.

In September 2023, the United States Food and Drug Administration released a draft guidance for comment about how informed client consent for companion animal clinical trials should be obtained (3). Frederick provides a review of this document, focused on the implications for those conducting veterinary clinical research. The guidance for consent timeframe, language, and specific elements will involve additional efforts by investigators to ensure adherence, yet might lead to increased owner compliance and higher enrollment in clinical studies with subsequent benefits for all.

The study of naturally occurring disease in populations of companion animals holds tremendous potential for informing the practice and progress in both veterinary and human medicine. Because data generated from these clinical trials may have an outsized impact on clinical treatments and outcomes, it is imperative that studies are conducted using the relevant, available best practice guidelines. The conversation toward the promotion of high quality veterinary clinical research is just beginning, and we hope that the manuscripts included in this Research Topic will promote further introspection that will benefit the animals, owners, researchers, and patients involved in future trials.
